# Influence of obstructive sleep apnea syndrome in the fluctuation of the submaximal isometric torque of knee extensors in patients with early-grade osteoarthritis

**DOI:** 10.1590/bjpt-rbf.2014.0106

**Published:** 2015-09-01

**Authors:** Andressa Silva, Marco T. Mello, Paula R. Serrão, Roberta P. Luz, Lia R. Bittencourt, Stela M. Mattiello

**Affiliations:** 1 Departamento de Fisioterapia, Universidade Federal de São Carlos (UFSCar), São Carlos, SP, Brasil; 2 Departamento de Esportes, Universidade Federal de Minas Gerais (UFMG), Belo Horizonte, MG, Brasil; 3 Departamento de Psicobiologia, Universidade Federal de São Paulo (UNIFESP), São Paulo, SP, Brasil

**Keywords:** osteoarthritis, sleep apnea syndromes, muscle strength, knee, rehabilitation

## Abstract

**OBJECTIVE:**

The aim of this study was to investigate whether obstructive sleep apnea (OSA) alters the fluctuation of submaximal isometric torque of the knee extensors in patients with early-grade osteoarthritis (OA).

**METHOD:**

The study included 60 male volunteers, aged 40 to 70 years, divided into four groups: Group 1 (G1) - Control (n=15): without OA and without OSA; Group 2 (G2) (n=15): with OA and without OSA; Group 3 (G3) (n=15): without OA and with OSA; and Group 4 (G4) (n=15) with OA and with OSA. Five patients underwent maximal isometric contractions of 10 seconds duration each, with the knee at 60° of flexion to determine peak torque at 60°. To evaluate the fluctuation of torque, 5 submaximal isometric contractions (50% of maximum peak torque) of 10 seconds each, which were calculated from the standard deviation of torque and coefficient of variation, were performed.

**RESULTS:**

Significant differences were observed between groups for maximum peak torque, while G4 showed a lower value compared with G1 (p=0.005). Additionally, for the average torque exerted, G4 showed a lower value compared to the G1 (p=0.036). However, no differences were found between the groups for the standard deviation (p=0.844) and the coefficient of variation (p=0.143).

**CONCLUSION:**

The authors concluded that OSA did not change the parameters of the fluctuation of isometric submaximal torque of knee extensors in patients with early-grade OA.

## Introduction

Sleep complaints in osteoarthritis (OA) patients have been recently reported in the literature^1^_,_ and some studies have described an increase in sleep disorders in this population, with Obstructive Sleep Apnea (OSA) being the most common and most frequent^1^^-^ 3.

It is estimated that in 2030, 20 to 30% of the world population will present some type of OA^4^, most frequently found in the population over 60 years^5^, particularly in the knee joint, which accounts for approximately 7% of cases^6^. OA is characterized by loss of articular cartilage and thickening of the joint capsule and is associated with changes in muscle function^7^, especially decreased quadriceps muscle strength^8^. Clinically, OA patients generally present with complaints of pain, fatigue, crepitus, limitations in performing activities of daily living^9^, and sleep complaints^1^.

The decrease in quadriceps muscle strength has been associated with functional changes and neuromuscular functional impairments, also due to OA^10^^,^ 11. Neuromuscular function plays an important role in knee joint stability, which involves muscle strength, coordination and the knee joint position sense^12^. Neuromuscular function arises from the integration of peripheral afferent signals of receptors located in the muscles, tendons, joint capsule, ligaments and menisci with motor efferent signals from supraspinal cortical areas providing coordination for the accurate activation and modulation of muscle force^12^.

The ability to produce and maintain a steady submaximal force production (i.e. *force steadiness)* has been previously studied^13^. Submaximal force assessment is a means to quantify deficits in neuromuscular control^14^.

Submaximal force reflects the deficits that might affect an individual's ability to reach the desired force and to successfully produce movement. This characteristic might be compromised in knee OA patients, and reports in the literature have suggested that this deficit might play an important role in knee OA pathogenesis^15^. However, the relationship between muscle strength and the fluctuation of the submaximal force is not fully understood. It has been suggested that the decrease in submaximal force fluctuation significantly contributes to the development and progression of knee OA^12^, but there is no current evidence to support this relationship^16^.

The aforementioned neuromuscular changes might affect the muscle strength of knee OA patients. Knowing that the presence of OSA in these patients leads to sleep deficit, these factors could influence the symptoms reported by the patients, such as pain and fatigue^17^. Moreover, the literature has shown that sleep deficit could induce muscle atrophy because of the decreases in anabolic hormones, such as testosterone, growth hormone and insulin-like growth factor 1 (IGF-1), and of the increases in catabolic hormones, such as myostatin and glycocorticoid^18^^,^ 19.

Considering that knee OA patients present changes in their sleep patterns, such as OSA, and both conditions compromise muscle function, this study's hypothesis was that the presence of OSA associated with OA would compromise the motor and functional capacities of patients and that OA alone would not. Therefore, this study aimed to assess if OSA affected the fluctuation of the submaximal isometric torque of knee extensors of patients with early-grade OA.

## Method

### Volunteers

**Figure 1.**
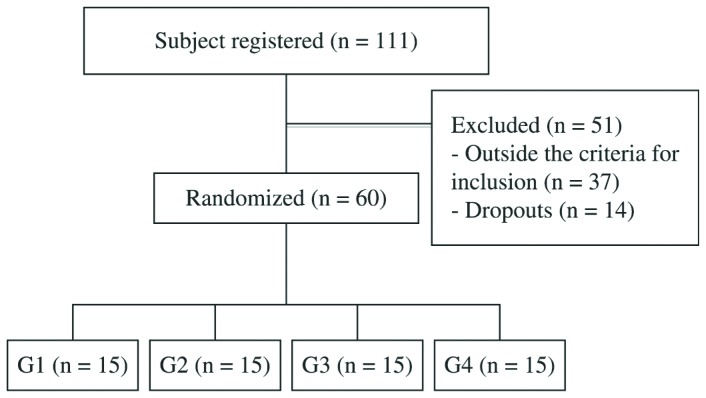
Flowchart of volunteers in this study.

The male volunteers were recruited via advertisements in print and electronic local media. Following the advertisement, a total of 111 individuals were initially enrolled, of whom 37 did not meet the inclusion criteria, and 14 dropped out (problems with the schedule of work (6), health problems (4), travel (2) and personal problems (2)) during the study (Figure 1). The study included men between 40 and 70 years of age diagnosed with knee OA according to the clinical criteria recommended by the *American College of Rheumatology* 20 and with severity grade II according to the Kellgren and Lawrence^21^ classification through X-ray examination. In addition, to be included in the study, the individuals could not have engaged in any regular physical activity in the last 6 months; could not have had any previous trauma, surgery or fracture of the lower limbs^10^; had not taken any pain medication and presented with normal resting and exercise electrocardiogram readings. All individuals underwent a polysomnography test and a sleep clinical.

Therefore, the final sample consisted of 60 volunteers separated into four groups based on the results of the data collected from the radiographs and sleep tests (see below): Group 1 (G1) (Control): without OA and without OSA (N=15); Group 2 (G2): without OA and with OSA (N=15); Group 3 (G3): with OA and without OSA (N=15); and Group 4 (G4): with OA and with OSA (N=15). The participants' characteristics are described in Table 1. The sample was homogeneous because the variables age [F _(3.56)_=0.559; p=0.644], height [F _(3.56)_=2.401; p=0.07], mass [F _(3.56)_=0.606; p=0.614] and body mass index (BMI) [F _(3.56)_=1.987; p=0.126] were not different between groups.

**Table 1.** Patient characteristics of the 4 groups.

	G1	G2	G3	G4
Age (years)	52.6±7.1	53.2±7.4	54.9±7.7	55.4±5.8
Stature (m)	175.6±6.4	174.9±6.9	173.3±8.8	169.1±6.5
Mass (kg)	81.5±9.7	84.9±16.9	78.7±11.1	82.5±12.0
BMI (kg/m^2^)	26.4±2.5	27.5±4.0	26.1±2.6	28.8±3.3
AHI (n/h)	3.1±2.0	29.4±30.8^*^	3.9±2.5&	24.6±20.4^*#^

Data are presented as means±SDs. One-factor ANOVA followed by Tukey's test. The assumed significance was alpha=0.05. *Different from G1; & Different from G2. #Different from G3. BMI: body mass index. AHI: apnea or hypopnea index; n/h: number/hour.

However, regarding the Apnea-Hypopnea Index (AHI) [F _(3.56)_=8.191; p=0.001; size effect=0.305; power=0.988], a higher AHI was detected in G4 and statistically differed from those in G1 (p=0.013) and G3 (p=0.017). A higher AHI was also observed in G2 and significantly differed from those in G1 (p=0.002) and G3 (p=0.002).

All of the participants signed an informed consent form, and the Universidade Federal de São Carlos (UFSCar) Ethics Committee, São Carlos, state of São Paulo - SP, Brazil (CEP #109/2011) approved the study.

### Procedures

The study was conducted at UFSCar, São Carlos city, and at the Centre for Studies in Psychobiology and Exercise (Centro de Estudos em Psicobiologia and Exercício - CEPE), São Paulo state, Brazil. First, the principal investigator interviewed the volunteers, and after, the resting and exercise electrocardiograms were scheduled with the cardiologist of the CEPE. If results of the electrocardiograms were normal, knee X-ray examinations were scheduled, and the results were analyzed by a rheumatologist of the CEPE. Then, each volunteer was referred to a sleep doctor who scheduled the polysomnography testing, and after the results were received, a new appointment was scheduled to diagnose OSA. Isokinetic testing was performed on volunteers only after all of these procedures were completed and the subjects were assigned to one of the 4 groups.

### Measurements

#### Radiographic assessment

Anteroposterior and mediolateral X-rays were taken from both knees of all participants. The criteria for the diagnosis of OA for Groups 3 and 4 was presence of well-defined osteophytes, without narrowing of the intra-articular space, classified as an early grade of the disease (grade II)^21^^,^ 22.

#### Polysomnography (PSG)

To record a full-night PSG, the *Embla* ^®^ S7000 (*Embla Systems, Inc., Reykjavik*, Iceland) device was used at the Sleep Laboratory (Sleep Institute [Instituto do Sono], São Paulo, Brazil). All recording sensors were attached to the patient in a non-invasive manner using adhesive tape or elastic bands. The following physiological variables were continuously and simultaneously monitored: 4-channel electroencephalogram (EEG) (C3-A2, C4-A1, O1-A2, O2-A1), 2-channel electrooculogram (EOG) (EOG-left-A2, EOG-right-A1), 4-channel surface electromyogram (muscle of the submentonian region, tibialis anterior muscle, region of the masseter muscle and 7^th^intercostal space) and 1-channel electrocardiogram (modified V1 derivation). Airflow detection was performed using 4 channels, namely one pair of thermal sensors (one channel) and nasal pressure (one channel), chest respiratory effort (one channel) and abdominal respiratory effort (one channel). Inductance plethysmography, snoring (one channel), position (one channel), oxygen saturation (SaO_2_) and pulse oximetry were also recorded. Sleep staging, awakenings and respiratory events were analyzed according to the criteria established in the *American Academy of Sleep Medicine Manual* 23. OSA was diagnosed by a sleep specialist. To be diagnosed with OSA for Groups 2 and 4, the volunteers presented with an AHI score from 5 to 15 and at least one complaint of snoring, sleepiness or a report of apnea, or an AHI score higher than 15 regardless of the symptoms^24^.

Based on the data from the radiographs and sleep tests, the volunteers were allocated to one of the 4 groups.

#### Assessment of maximum isometric peak torque and submaximal torque fluctuation

Maximum isometric peak torque and submaximal torque fluctuation of both knee extensors were assessed using an isokinetic dynamometer *(Biodex Multi Joint System 3, Biodex Medical Inc.,* Shirley, *New York, USA*). The isokinetic dynamometer was calibrated according to the manual provided by the manufacturer. Before the assessment, the volunteers performed a warm-up on a stationary bicycle for 5 minutes, with 75 W load and 20 km/h constant speed, followed by stretching of the lower limb muscles (i.e. quadriceps, hamstrings, gastrocnemius and soleus)^8^.

Isokinetic tests were conducted with the volunteer seated on the device, stabilized with the belts that crossed the trunk and pelvis. The dynamometer's mechanical axis of rotation was aligned with the lateral epicondyle of the femur, and resistance was applied distally at the ankle, 5 cm above the medial malleolus^8^. The volunteers were instructed to keep their arms crossed in front of the trunk during the test to avoid compensation.

The assessment of the maximal isometric torque of the knee extensors was performed with 60º flexion (0º full extension). Initially, the volunteers performed 5 maximal isometric contractions to determine maximum peak torque, with each contraction lasting 10 seconds and with 5 minutes of rest between contractions^10^. Before each assessment, the volunteers performed 3 submaximal contractions for familiarization with the procedures. During maximal contractions, a standardized verbal command was given to encourage the patients to reach maximum force in all contractions^8^.

For the torque fluctuation test, the target torque was set at 50% of maximum isometric peak torque^25^. During the submaximal isometric torque fluctuation test, the individuals received visual and verbal feedback. The participants were instructed to keep the produced torque line on the target torque line with the least possible oscillation for 10 seconds. Five attempts were made to maintain knee extensor torque, with 1 minute of rest between each attempt^26^.

#### Data processing

The isokinetic dynamometer data were collected with an acquisition frequency of 100 Hz and were analyzed using a routine programmed in *MatLab* ^®^ software (version 7.0.1, *MathWorks Inc., Natick, USA*). The variables used to express submaximal isometric torque fluctuation were standard deviation (SD) and the coefficient of variation (CV) (CV=SD/mean submaximal torque), calculated in an 8-second window^16^. Torque SD is an absolute measure of the submaximal torque fluctuation amplitude, and the torque CV was used as a relative fluctuation measure and was expressed as a percentage of the mean produced submaximal torque. The first two seconds of contraction were excluded to avoid the initial adjustment phase, as suggested by Lavender and Nosaka^27^. The assessments were always carried out in the period between the 2:00 pm and 6:00 pm and were executed by a researcher blinded.

### Statistical analysis

For the statistical analysis, the limb affected by OA or the most affected limb of the patients with bilateral OA was used^28^, and for the volunteers without OA, the limb to be statistically analyzed was selected by drawing lots.

The Shapiro-Wilk test was used for assessing data normality, and variables with non-parametric distributions (CV and SD) were normalized by Z score. Levene's test was used for assessing intra-group homogeneity. The analysis of the different parameters measured was performed using 1-factor analysis of variance (ANOVA; group factor) and Tukey's test for multiple comparisons using PASW 18 software. The results are expressed as means±SDs, and the alpha significance level was set at 0.05.

## Results

Table 2 shows the data regarding peak torque, exerted torque (50%), the CV and the SD of the submaximal isometric peak torque curve of the knee extensors. The authors detected a significant difference in isometric torque between groups [F _(3.56)_=5.288; p=0.003; size effect=0.224; power=0.913], and the value was smaller for G4 compared to G1 (p=0.005). There was also a significant difference in exerted torque at 50% between the groups [F _(3.56)_=3.594; p=0.019; size effect=0.164; power=0.763], with G4 showing a smaller value when compared with G1 (p=0.036). There were no differences in the CV [F _(3.56)_=1.881; p=0.143] and SD [F _(3.56)_=0.274; p=0.844].

**Table 2.** Peak torque during maximal isometric contraction, exerted torque, coefficient of variation and standard deviation during submaximal isometric contractions of the knee extensors of the 4 groups.

	G1	G2	G3	G4
Peak Torque (Nm)	226.0±47.3	217.3±35.86	190.6±28.3	181.4±36.4^*^
Exerted Torque (Nm)	117.4±19.9	114.3±21.1	102.4±15.4	95.8±21.9^*^
Standard Deviation	3.2±1.3	3.7±2.3	3.5±2.8	3.1±1.1
Coefficient of Variation	3.2±1.2	4.2±3.0	4.1±2.8	3.4±1.0

Data presented as means ± SDs. One-factor ANOVA followed by Tukey test's. The significance used was alpha=0.05. *Different from G1.

## Discussion

Considering that patients with both OA and OSA might present changes in muscle function, the results of this study showed that maximal isometric torque and exerted torque were altered in patients with both OSA and grade II OA; however, the submaximal isometric torque fluctuation of the knee extensors remained unchanged. This study identified a smaller maximum isometric peak torque and exerted torque (50%) in the group with both OA and OSA (G4); however, there were no differences between groups in terms of CV and SD, demonstrating that the pattern of muscle strength might be altered in early-grade OA associated with OSA, but still without neuromuscular control impairment during submaximal isometric torque of the knee extensors.

This study was the first to investigate whether OSA could affect submaximal isometric torque fluctuation in patients with knee OA. OSA and OA incidence might be associated with the fact that the prevalence of both diseases increase with aging^29^. Additionally, it has been demonstrated that poor sleep quality alters pain and fatigue symptoms in OA patients, which could change muscle strength. Recently, it has been shown that sleep debt could induce muscle atrophy^18^^,^ 19 because it leads to metabolic changes in the muscle, affecting muscle recovery due to increased stimulation of protein degradation, by which protein synthesis causes muscle atrophy. Therefore, in this study, decreased maximal isometric torque and exerted torque (50% of maximal isometric torque) was observed in G4 (OA and OSA) when compared with the control group (G1), demonstrating that the patients who exhibited changes in their sleep patterns associated with early-grade knee OA might present impairments in both maximal isometric torque and exerted torque.

This decrease in isometric torque in OA patients was in agreement with other studies that have also reported decreased isometric torque in knee OA patients^30^^-^ 31; however, these studies assessed OA patients at all stages of the disease. The present study assessed patients with early-grade knee OA, who already presented with decreased isometric muscle strength of the quadriceps, which has been highlighted as a risk factor for the onset of certain symptoms, such as pain^32^.

The control of submaximal muscle force production has been considered important in activities of daily living, such as walking, transfers, and sitting and standing^10^. Tracy and Enoka^33^ stated that an optimal submaximal muscle force appeared to be an indicator of good neuromuscular function, leading to better capacity to control and coordinate knee movement. Conversely, worse neuromuscular function has been related to increases in the harmful forces applied to the knees, which might contribute to OS development and to its progression in the long term^16^.

Studies that have assessed submaximal torque fluctuation in different joints have shown that the SD and CV variables are the most representative 0of the fluctuation^34^^,^ 35. In the present study, SD and CV did not differ between groups, and these results corroborate with the findings of Hortobágyi et al.^10^, who assessed 20 individuals with knee OA and 20 without knee OA and concluded that, although the OA group had worse physical function than the healthy group, the submaximal isometric torque fluctuation of the knee extensors remained unchanged in OA patients. On average, the patients included in the present study showed a submaximal isometric torque fluctuation of the knee extensors (Table 2) similar to that reported by Hortobágyi et al.^10^; however, comparisons between the two studies should be made carefully, as they used different assessment methods. In the present study, a target force of 50% of the maximal isometric torque was used, whereas Hortobágyi et al.^10^ used a target force of 50 to 100 N for all participants.

Changes in motor control, such as the submaximal force fluctuation, could affect knee mechanics during gait and, thus, could be associated with the knee adduction moment, as this moment could be an indirect predictor of the load applied to the medial compartment of the knee during gait^36^. However, the study by Sørensen et al.^16^ investigated the relationship between quadriceps isometric force fluctuation and the knee adduction moment in the frontal plane when studying the gaits of 41 patients with different grades of knee OA and found no association between quadriceps isometric force fluctuation and the knee adduction moment.

However, aging could modify motor control and submaximal force because older people have demonstrated mixed results regarding the effects of age on submaximal force fluctuation of the knee extensors, as observed in one study that reported a decrease in the submaximal torque fluctuation^33^, while others found no change^10^^,^ 37.

Considering that pain perception might be altered by sleep disorders, as demonstrated in studies with animals and humans^38^^-^ 40 with OA, this change in pain is a factor that could lead to a change in motor performance, increasing torque fluctuation, as observed in an experimental pain model^13^. However, it is important to note that the knee OA patients of this study did not report pain during submaximal isometric torque fluctuation assessment because the increase in the afferent signals sent by the pain receptors could reduce proprioceptive afference and, thus, could modify motor control due to the pain^41^.

The study of Bandholm et al.^13^, which assessed pain effects on patients with subacromial impingement syndrome, reported a deficit in the submaximal concentric torque fluctuation; however, they did not find a difference in the submaximal isometric torque fluctuation. The same behavior was observed in the study of Zanca et al.^35^, which reported no difference in submaximal isometric torque fluctuation in patients with subacromial impingement syndrome. However, these studies suggest that higher levels of shoulder pain could lead to higher submaximal torque fluctuation, thus corroborating our results.

The present study analyzed four groups in an attempt to elucidate the relationship between sleep disorder and OA, and in the results, it was observed that patients in the OSA and OA groups (G4) presented losses in torque when compared with the healthy individuals in the control group (G1) (without both OA and OSA). However, the presence of OSA alone (G2) or OA alone (G3) did not result in differences in functional or motor capacities when compared with the healthy group (G1). Therefore, it was not possible to say whether OSA affected OA or vice-versa because the G2 and G3 groups showed no differences between them. However, in a qualitative analysis, the G3 group was found to exhibit a smaller torque than G2, indicating that OA modified torque more than OSA. However, when combined, OSA and OA negatively modified the knee's functional capacity, as observed in G4 (with OA and with OAS).

It is worth noting that this study was the first to investigate motor function and control in OA and OSA; thus, the mechanisms involved have not yet been elucidated. Longitudinal and cohort studies should be strongly encouraged to better understand OSA and OA.

This study has some limitations, such as the lack of assessment of electromyographic activity in knee extensors during submaximal isometric torque fluctuation assessment, which would allow better understanding the activation pattern of the extensor muscles because there were no deficits in neuromuscular function in participants with knee OA, as assessed by electromyography^42^. Another limitation is the lack of follow-up using a daily sleep questionnaire, which would provide a daily assessment of the sleep routine of the volunteers during the study.

Based on this study's results, it can concluded that OA associated with OSA modified maximal isometric torque and exerted torque; however, these conditions did not change the submaximal isometric torque fluctuation of the knee extensors, indicating that, although OSA negatively affected the musculoskeletal system of patients with grade II knee OA, this syndrome did not alter the neuromuscular control in this population.
